# Challenges With Long-Term Care Placement in Pediatric Psychiatry During COVID-19: A Case Series From Inpatient Unit

**DOI:** 10.7759/cureus.19947

**Published:** 2021-11-27

**Authors:** Jaskaranpreet Kaur, Ankit Jain

**Affiliations:** 1 Otolaryngology - Head and Neck Surgery, Dayanand Medical College (DMC), Ludhiana, IND; 2 Psychiatry and Behavioral Health, Penn State College of Medicine, Hershey, USA

**Keywords:** mental illness, children and families, children and youth services, inpatient psychiatry, foster care, long-term care, covid-19

## Abstract

A large number of children and youth have been affected worldwide during the coronavirus disease 2019 (COVID-19) pandemic. Apart from the obvious medical and financial impacts of the COVID-19 pandemic, there has been an immense amount of psychosocial impact specifically on children and adolescents ranging from parents losing their jobs leading to financial hardships to school closure leading to not having significant interaction with their peers and having to spend more time at home which often leads to psychosocial conflicts. According to the literature review, in recent times, there has been worsening of mental health illnesses, increased suicide attempts, and severity of suicide attempts which has led to more visits to the hospitals and clinics with psychiatric concerns. There has been a pattern of increasing patient acuity in inpatient psychiatric units, more specifically in the pediatric units. The average length of stay for inpatient units has been increasing in the pediatric psychiatric units and an unfortunate downside of which has been noted to be increased lag time for placements in foster care systems or residential treatment centers in recent times. Here, we present cases of three teenagers who faced significant challenges in obtaining placement to foster care systems or residential treatment centers in the context of longer wait times experienced for intensive-out-of-home treatment services during the COVID-19 pandemic and discuss potential solutions to this issue.

## Introduction

More than 5.1 million people have died due to coronavirus disease 2019 (COVID-19) caused by the severe acute respiratory syndrome coronavirus worldwide. More than 1.5 billion children and youth have been affected in 188 countries so far during the COVID-19 pandemic [1]. COVID-19 deaths disproportionately occur among adults, which has led to many children losing their parents and caregivers. Apart from the obvious medical and financial impacts of the COVID-19 pandemic, there has been an immense amount of psychosocial impact specifically on children and adolescents ranging from parents losing their jobs leading to financial hardships to school closure leading to not having significant interaction with their peers and having to spend more time at home which often leads to psychosocial conflicts. With increased vaccinations, many schools are getting back to in-person schooling or a combination of virtual and in-person schooling; due to increased social isolation over the past two years, there has been a significant impact on the normal developmental trajectory for children and adolescents across the world including the United States. There has been an increased number of psychiatric presentations to emergency rooms as well as to hospitals in the context of worsening mental health illnesses and increased suicide attempts and severity of the suicide attempts over the past two years [2-4]. There has been a pattern of increasing patient acuity in inpatient psychiatric units, more specifically in the pediatric units. The average length of stay for inpatient units has been increasing in the pediatric psychiatric units and an unfortunate downside of which has been noted to be increased lag time for placements in foster care systems or residential treatment centers in recent times. There has been a steady increase in residential treatment facility referrals during the pandemic, making it harder to find a proper step-down option for the patient who has been treated on the inpatient units. Outside-of-the-home, children often require extra care, often due to difficult family and psychosocial challenges. More than half of children in out-of-home care have significant mental health difficulties [5]. Foster parents are sometimes forced to rethink their foster care offer due to stress and school closures, resulting in a placement disruption [6]. Additionally, foster care providers are more susceptible to COVID-19. They are typically old, often over 65 years, predisposing them to increase the risk of contracted COVID-19 infection from children that they take care of who were often not vaccinated up until recently [7]. The number of children in the United States placed in foster care has been estimated to be between 5% and 6%, with about 10% of African American children and 15% of Native American children out of all the children in the foster care system [8]. Residential facilities face several new challenges like increased staff absences, overcrowded facilities, social isolation, and low support from educational, social work, and medical services standpoints.

Here, we present cases of three teenagers who faced significant challenges in obtaining placement to foster care systems or residential treatment centers in the context of longer wait times experienced for intensive-out-of-home treatment services during the COVID-19 pandemic.

## Case presentation

Case 1

The patient is a 15-year-old transgender female to male patient with a history of post-traumatic stress disorder (PTSD), disruptive mood dysregulation disorder (DMDD), and borderline personality traits. The patient has a history of multiple previous psychiatry hospitalizations for concerns related to urges to self-harm and having suicidal ideations, diabetes mellitus type 1 with well-controlled blood sugar on their insulin regimen, no other medical history was noted. The patient was started on duloxetine 30 mg daily which was gradually titrated up to 90 mg daily to address depression as well as issues with chronic pain, which are mainly abdominal in the location with some diffuse body pain and melatonin 5 mg as needed for insomnia. The patient has been waiting in the inpatient unit for more than four months pending placement at a community residential rehabilitation (CRR) host home. The patient is having trouble finding a placement given his specific needs including medical management issues as well as increased difficulty in placing patients during the COVID-19 pandemic. The patient has been denied at multiple CRR host home facilities given their specific needs. Child and Youth Services is currently involved in looking for placement options for the patient, who is hoping to get discharged soon.

Case 2

The patient is a 13-year-old white female with a history of post-traumatic stress disorder, unspecified mood disorder, and generalized anxiety disorder, who was admitted to the inpatient unit in the context of worsening suicidal ideations and depressions because of increased conflicts at home with her parents. The patient was successfully treated on the inpatient unit with sertraline which is started at 25 mg per day and was optimized to 150 mg per day. The patient was also started on buspirone at a dose of 5 mg twice a day which was optimized to 15 mg thrice a day. The patient was also put on topiramate starting at a dose of 50 mg which was gradually titrated up to 100 mg per day to address her migraines. The patient was not agreeable to going back home because of increased conflicts that she reported having with her mother at home. Despite multiple family meetings and various attempts, there was not an easy resolution to the issues that the patient had at home. Finally, a referral to CRR host home placement was made. It took roughly two months for the patient to finally get placed at a CRR host home despite meeting all the criteria for the same. This was another case that re-emphasized the difficulty with long-term placements that psychiatrists and the treatment team in the inpatient psychiatry unit have been facing to help place patients, long after they have met the goals for inpatient treatment.

Case 3

The patient is a 17-year-old white male with a history of autism spectrum disorder (ASD), major depressive disorder (MDD), and attention deficit hyperactivity disorder (ADHD), who was admitted to the inpatient unit in the context of having increased arguments and verbal altercation with his siblings at home. During the course of the hospital stay, the patient's medications were optimized, risperidone 0.5 mg per day was initiated which was gradually titrated up to 2 mg twice daily which the patient tolerated well. The patient was also started on long-acting guanfacine 1 mg per day which was gradually titrated up to 4 mg per day by the time of discharge. The patient was also started on extended-release methylphenidate which was gradually titrated up to 54 mg per day by the time of discharge. After making all of these medication changes, significant improvement in the patient's impulsivity, mood lability, and hyperactivity was noted. The patient was noted to participate well in the groups and follow staff directions well for the most part. Because of ongoing difficulties with his interactions with his siblings and foster siblings at home, the patient's parents voiced reluctance to take the patient upon discharge and requested a long-term placement. Given the age of the patient, a residential treatment center was not an option and thus, a referral for foster home placement was made. Despite multiple meetings with various foster homes, Child and Youth Services as well as with the patient's parents, the patient was not able to find an appropriate placement that would suit his needs. After waiting in the inpatient unit for approximately three months, the patient was finally administratively discharged back to the care of his parents, long after the patient had already met his treatment goals in the inpatient psychiatry unit.

## Discussion

Due to ongoing school closure for the entirety of the pandemic with some relief more recently, families of the children are often burdened with increased parenting demands which place children at increased risk. Foster parents may have to revisit their decisions of wanting to support children because of increased financial and social burden, because of children not being in school leading to further placement disruptions [9]. Family support systems such as grandparents and siblings of the parents may also have decreased ability to help foster the children in need because of a multitude of challenges that the pandemic has brought with it including job losses, financial burden, and increased emotional stress that the parents are facing on a day-to-day basis.

A significant increase in referral for long-term placements has been noted across the psychiatric facilities in the United States during the COVID-19 pandemic. COVID-19 pandemic has created its own sets of challenges for children being treated in psychiatric units and making it a lot more challenging to find the placement with strict quarantining, masking, and COVID-19 testing guidelines, which individual residential treatment centers often have [2,10]. Another downside of school closure over the past one to 1.5 years has been reduced access to healthcare and therapy services that are available to the kids in need of behavioral health treatment which involves frequent contact with the therapist as well as regular follow-up. According to a study, 58% of mental health needs for adolescents came through the educational setting with higher rates among minority students and low-income groups [11].

Several countries have had significant delays in reporting concerns regarding children's safety and well-being. Global estimates for depression and anxiety, the two most common childhood mental disorders, were 8.5% and 11.6%, respectively, before COVID-19 [12,13]. In a meta-analysis by Racine et al., the prevalence of clinically significant depression (23.8%) and anxiety (19%) for children and adolescents was significantly higher than before the pandemic [14]. When schools were closed, accessing community-based services was difficult due to financial constraints or a lack of pre-existing relationships with caregivers. In this context, it is essential to promote the use of existing school-based resources. While schools have tended to transition rapidly to distance learning during the COVID-19 pandemic, school counselors have spent less time directly working with students due to administrative demands during rapid school pivots [15]. Mental health workers have an opportunity to strengthen school funding by responding to the consequences of the COVID-19 pandemic. Besides in-person, there may be opportunities to enhance the relationship between school-based mental health support and community mental health support while students utilize distance learning and return to class. There are several ways to implement this process, including creating regular channels of connection between students and school counselors (for example, through screenings or regular check-ins) and experimenting with novel and effective forms of mental health support, such as one-time online interventions [16]. Adults in the family also suffered mental stress in the pandemic which subsequently negatively affected the children’s mental health. Telehealth services could be used by adults as well as children to decrease the impact of stress from the global impact of the COVID-19 pandemic. It is paramount to utilize the services based in the community to help address the ever-increasing mental health needs of the children and their families in the context of this pandemic. Higher emphasis on utilizing services such as family-based services and community treatment options needs to be utilized to decrease a burden on our already crumbling and heavy burden foster care system. Timely interventions and the use of resources such as in-home therapy, family-based services, community mental health clinics can be extremely crucial in avoiding extended length of stay in the inpatient unit looking for referrals to the foster care systems. In the above-presented cases, successful placements stemmed from the prompt response by children and youth services caseworkers and sustained diligence from the inpatient coordination team. 

Increased access to telepsychiatry services for both pharmacological management as well as psychotherapeutic options is going to be crucial to help to decrease increasing patient visits to emergency rooms for behavioral health needs in her pediatric population. Furthermore, timely follow-ups and medication refill by ensuring regular follow-ups are equally crucial in avoiding unnecessary emergency room visits as well as admissions to the inpatient psychiatry unit, which can often turn into a referral for long-term placement. It is also important to note that placements in long-term facilities do not come without inherent risks with the placements in the context of worsening behavioral issues, along with potential increased exposure to COVID-19 infection during this pandemic [17]. While the pediatric population has predominantly been unaffected during this pandemic, there is a lot that is unknown about the complications and now emerging neuropsychiatric sequel of COVID-19 infection [10,18].

## Conclusions

Even before the pandemic, children and families struggled to find placement in group homes. Children with special needs are experiencing an admission crisis due to an exponential decline in in-home visits from psychiatrists, fewer children with mental illness are visiting clinics because of fear of infection, longer clinic closures, increased family abuse, and exaggerated symptoms. In the wake of the pandemic, there has been an increased referral to inpatient units seeking long-term placements for children and adolescents struggling with worsening mental health issues. To minimize the impact of the placement process on children already suffering from psychopathological conditions in the future, we must focus on the solution to handle this crisis judiciously. Measures should be taken to increase telehealth service as it is cost- and time-effective. Increased access to telehealth community-based treatment options such as in-home services will be extremely crucial. Parents should take an active role in understanding the mental health needs of their children and themselves. There is also a need for increased funding foster care systems and child and youth services who are burdened with the task of providing placement options with limited resources at their disposal.
